# Clinical significance of the combination of preoperative SUVmax and CEA in patients with clinical stage IA lung adenocarcinoma

**DOI:** 10.1111/1759-7714.14599

**Published:** 2022-08-12

**Authors:** Asato Hashinokuchi, Naoki Haratake, Tomoyoshi Takenaka, Kyoto Matsudo, Taichi Nagano, Kenji Watanabe, Keisuke Kosai, Yuka Oku, Yuki Ono, Shinkichi Takamori, Mikihiro Kohno, Shingo Baba, Kousei Ishigami, Tomoharu Yoshizumi

**Affiliations:** ^1^ Department of Surgery and Science, Graduate School of Medical Sciences Kyushu University Fukuoka Japan; ^2^ Department of Clinical Radiology, Graduate School of Medical Sciences Kyushu University Fukuoka Japan

**Keywords:** carcinoembryonic antigen (CEA), lung adenocarcinoma, standardized uptake value (SUV)

## Abstract

**Background:**

Preoperative maximum standardized uptake value (SUVmax) of 2‐[18F]‐fluoro‐2‐deoxy‐D‐glucose positron emission tomography and serum carcinoembryonic antigen (CEA) have been reported as prognostic factors for lung adenocarcinoma. However, the significance of combined SUVmax and CEA in early‐stage lung adenocarcinoma is not well known.

**Methods:**

We retrospectively evaluated the relationship between the combination of SUVmax and CEA and the prognosis of 410 patients with clinical stage IA lung adenocarcinoma who underwent resection. The cutoff values for SUVmax and CEA were determined by receiver operating characteristic curve analysis, and patients were categorized into high SC (SUVmax and CEA) group (SUVmax ≥2.96 and CEA ≥5.3), moderate SC group (either SUVmax <2.96 and CEA ≥5.3 or SUVmax ≥2.96 and CEA <5.3) and low SC group (SUVmax <2.96 and CEA <5.3).

**Results:**

Kaplan–Meier curve analysis showed that patients with clinical stage IA lung adenocarcinoma in the high SC group had significantly shorter overall survival (OS) and recurrence‐free survival (RFS) than the other groups (*p* = 0.011 and *p* < 0.0001, respectively). Multivariate analysis showed that high SC was an independent prognostic factor of OS (*p* = 0.029) and RFS (*p* < 0.0001).

**Conclusions:**

High values of SUVmax and CEA were associated with poor OS and RFS in patients with stage IA lung adenocarcinoma. Simultaneous evaluation of SUVmax and CEA may be an effective prognostic marker to determine the optimal treatment strategy of early‐stage lung adenocarcinoma.

## Graphical abstract

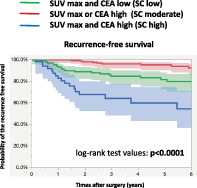

## INTRODUCTION

Improvements in diagnostic and screening techniques have led to the increased detection of early‐stage lung cancer. Surgery is currently the best therapeutic modality for patients with early‐stage lung cancer, and lobectomy has been established as the standard procedure. However, recent reports demonstrated the efficacy of segmentectomy for small sized lung cancer.^1^, ^2^ A multicenter, randomized, controlled, phase 3 trial (JCOG0802/WJOG4607L) showed the superiority of segmentectomy over lobectomy in overall survival (OS) for patients with clinical stage IA non‐small cell lung cancer (NSCLC) (tumor diameter ≤2 cm and consolidation/tumor ratio >0.5).^3^, ^4^ Thus, segmentectomy may become the one of the standard surgical treatment options for small‐sized lung adenocarcinoma. However, this study also reported that locoregional relapses occurred more frequently in segmentectomy than in lobectomy.^4^ Whether all patients with small‐sized lung adenocarcinoma require segmentectomy remains unclear. Accurate prognostic factors may be needed to determine indications for segmentectomy.

In a previous study, the overall survival rate at 5 years was 83.5% in patients with clinical stage IA NSCLC. However, the overall survival rate at 5 years for stage IA1 was 91.6%, 81.4% for stage IA2 and 74.8% stage IA3.^5^ In addition, the presence of a ground‐glass opacity component was reported as a prognostic factor, and the 5‐year overall survival in patients of clinical stage IA with a ground‐glass opacity was 95.1% compared with 81.1% in patients with a solid component.^6^ Therefore, the prognosis of patients with clinical stage IA varies, and further research to identify appropriate prognostic factors is important.

Several prognostic factors of lung adenocarcinoma are important for giving useful additional data to select the optimal treatment strategy for patients. Invasive component size, visceral pleural invasion and blood and lymphatic vessel invasion in pathology were reported as risk factors of recurrence for patients with stage I lung adenocarcinoma.^7^, ^8^ Locoregional recurrence was also frequently observed in patients with a radiological pure solid NSCLC on computed tomography (CT) who underwent segmentectomy.^9^ Spread through air spaces in resected lung adenocarcinoma was reported to correlate with poor recurrent‐free survival (RFS).^10^ Several studies have reported that high level of carcinoembryonic antigen (CEA) is associated with poor prognosis in patients with lung adenocarcinoma.^11^, ^12^, ^13^, ^14^, ^15^ In addition, high maximum standardized uptake value (SUVmax) of 2‐[18F]‐fluoro‐2‐deoxy‐D‐glucose positron emission tomography (FDG‐PET) correlates with more advanced disease and high‐risk features in patients with early‐stage lung adenocarcinoma.^16^ However, few studies have assessed whether the combination of SUVmax and CEA is a prognostic factor of clinical stage IA lung adenocarcinoma.

The aim of this study was to assess the significance of preoperative SUVmax and CEA for the prediction of prognosis in patients with clinical stage IA lung adenocarcinoma. These findings may contribute to determination of an optimal treatment strategy for patients with small sized lung adenocarcinoma.

## METHODS

### Patients and samples

We retrospectively identified and enrolled 410 patients with preoperative clinical stage IA lung adenocarcinoma who underwent surgery between 2009 and 2018 at Kyushu University Hospital. No patients had received preoperative radiotherapy or chemotherapy. We excluded patients with pathological stage IV lung adenocarcinoma. The clinical TNM stage was diagnosed on the basis of chest and upper abdomen CT, brain magnetic resonance imaging (MRI) or CT and FDG‐PET before surgery. Clinical stage was classified by FDG‐PET/CT; N0 stage was defined as mediastinal and hilar lymph nodes less than 1 cm in the short‐axis diameter and no FDG uptake compared with the background activity of the surrounding mediastinal or lung tissues. The SUVmax of the primary tumors was measured in all patients. The SUV is the ratio of the image‐derived radioactivity concentration in tissue to the concentration of radioactivity in the whole body. The serum level of CEA was measured by an enzyme‐linked immunosorbent assay before surgery and the cutoff value was 3.2 ng/ml. The pack‐year is a unit for measuring the amount a person has smoked over a long period of time. It is calculated by multiplying the number of packs of cigarettes smoked per day by the number of years the person has smoked.

At our institution, if a tumor was pure GGO or GGO‐dominant, the patients received sublobar resection, whereas major lung dissection with systematic or selective lymph node dissection was warranted for part‐solid or solid tumors. Some patients who were older, had a high cardiopulmonary risk or could not tolerate lobectomy for any other reason received sublobar resection. The histological types of lung adenocarcinoma were classified following the International Association for the Study of Lung Cancer/American Thoracic Society/European Society of Thoracic Surgeons classification^17^ and the TNM stage was determined using the eighth edition of the TNM classification.^18^ Written informed consent to access medical records was obtained from each patient. This study was approved by the institutional review of board of Kyushu University Hospital (IRB number: 2019–232).

### FDG‐PET

In each patient, 185 MBq FDG was intravenously administered after fasting for at least 4 h. Scans were conducted from the middle of the thigh to the top of the skull 60 min after FDG administration. FDG‐PET/CT images were obtained using an integrated PET/CT scanner (Discovery STE; GE Medical Systems, Milwaukee, WI, USA) or Biograph mCT (Siemens Medical Solutions, Erlangen, Germany). All emission scans were performed in three‐dimensional mode, and the acquisition time per bed position was 3 min for Discovery STE and 2 min for Biograph mCT. We reconstructed PET images using the ordered‐subset expectation–maximization method (VUE Point Plus) with two full iterations of 28 subsets for the Discovery STE and iterative True‐X algorithm and TOF (Ultra HD‐PET) with two full iterations of 21 subsets. The True‐X algorithm incorporates an additional specific correction for the point‐spread function. The full‐width at half‐maximum values of the Discovery STE and Biograph mCT was 5.2 and 4.4 mm, respectively. A low‐dose 16‐slice CT (tube voltage 120 kV; effective tube current 30–250 mA, Discovery STE) and a low‐dose 32‐slice CT (tube voltage 120 kV; use of angular and longitudinal dose modulation, CAREDose4D®, Biograph mCT) from the vertex to the proximal thigh were performed for attenuation correction and for determining the precise anatomic location of lesions before acquisition of PET images. CT scans were reconstructed by filtered back projection into 512 × 512 pixel images with a slice thickness of 5 mm to match the PET scan. FDG uptake in lesions was evaluated using SUVmax, calculated on a dedicated workstation for each scanner.

### Follow‐up

After surgical resection, routine check‐ups (including a physical examination, blood tests including serum CEA and chest x‐ray) were performed at 3‐month intervals for the first 3 years and at 6‐month intervals thereafter. The cutoff date was April 31, 2020. CT was performed twice each year for the first 3 years and at 1‐year intervals thereafter. Adjuvant chemotherapy was applied to patients <76 years old with pathological stage IB to IIIA and performance status 0 and 1. The regimen for patients with pathological stage IB adenocarcinoma was uracil‐tegafur, and patients with stage IIA to IIIA received a platinum‐based combined regimen. If recurrent disease was suspected, further evaluations such as MRI and FDG‐PET were performed. Recurrent NSCLC was diagnosed on the basis of physical examinations and diagnostic imaging findings consistent with recurrent disease. When clinically feasible, diagnoses were histologically confirmed. The date of recurrence was defined as the date when recurrence was histologically proven or, in cases that were diagnosed by clinical evidence, when recurrent disease was recognized by the attending physician. RFS was defined as the period from the operation to the relapse of the disease and OS was defined as the period from the operation to mortality or censored observation at the cutoff day.

### Statistical analysis

**FIGURE 1:**
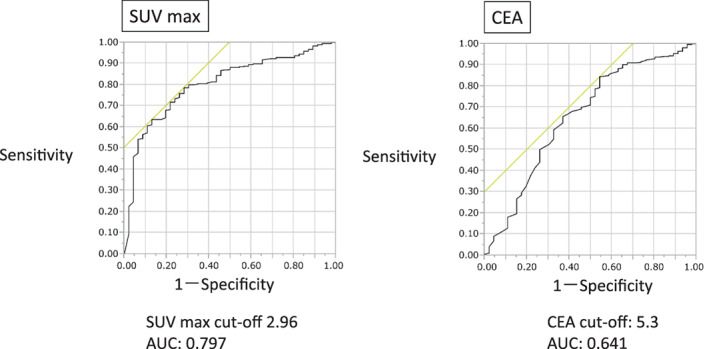
Receiver operating characteristic curve for maximum standardized uptake value (SUVmax) and carcinoembryonic antigen (CEA) in patients with preoperative clinical stage IA lung adenocarcinoma. The optimal cutoff points were 2.96 for SUVmax and 5.3 for CEA

An unpaired *t*‐test was used to compare the factors. Values for OS and RFS were calculated by Kaplan–Meier estimation methods using a log‐rank test. The receiver operating characteristic (ROC) curves of the SUVmax and CEA were used to predict recurrence. The values for the area under the ROC curve were compared and the cutoff values were determined. The optimal cutoff points were 2.96 for SUVmax and 5.3 ng/ml for CEA (Figure 1). Patients were categorized into the high SC (SUVmax and CEA) group (SUVmax ≥2.96 and CEA ≥5.3), moderate SC group (either SUVmax <2.96 and CEA ≥5.3 or SUVmax ≥2.96 and CEA <5.3) and low SC group (SUVmax <2.96 and CEA <5.3). Univariate and multivariate analyses with Cox proportional hazards regression analysis were performed to assess the relationship between OS and RFS with clinical features. We used the backward elimination method for multivariate Cox proportional hazards regression analysis; the model was run with all variables and the variable with the highest *p*‐value. A *p*‐value less than 0.05 was considered statistically significant. All statistical analyses were performed using JMP software program, version 16.0.

## RESULTS

### Clinicopathological characteristics of patients

Table 1 shows the clinicopathological characteristics of the 410 patients with clinical stage IA lung adenocarcinoma who underwent complete resection. The median age of the patients was 69.0 years (range: 33 to 88); 200 (48.8%) patients were male and the pack‐year index <20 was 287 (70.0%). The median CEA was 2.6 (range: 0.4 to 129.8) and the median SUVmax was 2.2 (range: 0 to 20.2). Regarding preoperative clinical staging, 146 (35.3%), 177 (43.2%) and 87 (21.4%) patients had stage IA1, IA2 and IA3, respectively. The most frequently performed operation was pulmonary lobectomy (286, 69.8%), followed by partial resection (69, 16.8%) and segmentectomy (55, 13.4%). Video‐assisted thoracoscopic surgery (VATS) (259, 63.2%), complete VATS (90, 22.0%) and open thoracotomy (61, 14.8%) were performed.

**TABLE 1:** Clinicopathological characteristics of patients with clinical stage IA lung adenocarcinoma who underwent resection (*n* = 410)

Characteristic	n (%)
Age (years; range)	69 (33–88)
Sex	
Male	200 (48.8%)
Female	210 (51.2%)
Smoking status	
<20 PYI	287 (70.0%)
≥20 PYI	123 (30.0%)
T‐primary tumor	
T1mi	56 (13.7%)
T1a	90 (21.9%)
T1b	177 (43.2%)
T1c	87 (21.2%)
TNM staging (clinical)	
IA1	146 (35.6%)
IA2	177 (43.1%)
IA3	87 (21.2%)
Findings on CT	
Part‐solid	220 (53.7%)
Solid	190 (46.3%)
CEA (median; range)	2.6 (0.4–129.8)
SUVmax (median; range)	2.2 (0–20.2)
Operation method	
Partial resection	69 (16.8%)
Segmentectomy	55 (13.4%)
Lobectomy	286 (69.8%)
TNM staging (pathological)	
0	11 (2.7%)
IA1	95 (23.3%)
IA2	168 (41.0%)
IA3	48 (11.7%)
IB	48 (11.7%)
II	19 (4.6%)
III	21 (5.1%)
Pathological classification	
AIS	17 (4.1%)
MIA	14 (3.4%)
Acinar	55 (13.4%)
Lepidic	23 (5.6%)
Papillary	252 (61.5%)
Micropapillary	4 (0.98%)
Solid	28 (6.8%)
Invasive mucinous	10 (2.4%)
Other	7 (1.7%)

Abbreviations: AIS, adenocarcinoma in situ; CEA, carcinoembryonic antigen; CT, computed tomography; MIA, minimally invasive adenocarcinoma; PYI, pack‐year index; SUVmax, maximum standardized uptake value; T, tumor; N, lymph node; M, metastasis.

Table 2 showed the clinicopathological factors of patients in the high SC group (*n* = 48, 11.7%), the moderate SC group (*n* = 148, 36.1%) and the low SC group (*n* = 214, 52.2%). High SC was markedly associated with sex (male), smoking history (pack‐year index ≥20), findings on CT (C/T ratio >0.5), clinical stage (IA3), histological subtype (except for adenocarcinoma in situ/minimally invasive adenocarcinoma) and operation method (lobectomy), but it was not associated with age (≥65 years).

**TABLE 2:** Association between SUVmax and CEA and clinicopathological factors in patients with clinical stage IA lung adenocarcinoma who underwent surgery

Factors	High	Moderate	Low	*p*‐value
	SUVmax + CEA	SUVmax + CEA	SUVmax + CEA	
	(*n* = 48)	(*n* = 148)	(*n* = 214)	
Age				
<65 years	8 (16.7%)	46 (31.3%)	65 (30.4%)	*p* = 0.1282
≥65 years	40 (83.3%)	102 (68.7%)	149 (69.6%)	
Sex				
Male	32 (66.7%)	78 (52.7%)	90 (42.1%)	*p* = 0.0042
Female	16 (33.3%)	70 (47.3%)	124 (57.9%)	
Smoking status				
<20 PYI	22 (45.8%)	78 (52.7%)	149 (69.6%)	*p* = 0.0004
≥20 PYI	26 (54.2%)	70 (47.3%)	65 (30.4%)	
CT ratio				
<0.5	45 (93.8%)	135 (91.2%)	133 (62.2%)	*p* < 0.0001
≥0.5	3 (6.2%)	13 (8.8%)	81 (37.8%)	
Clinical stage				
IA1	4 (8.3%)	23 (15.5%)	119 (55.6%)	*p* < 0.0001
IA2	21 (43.8%)	75 (50.7%)	81 (37.9%)	
IA3	23 (47.9%)	50 (33.8%)	14 (6.5%)	
Pathological stage				
0	0 (0.0%)	0 (0.0%)	11 (5.1%)	*p* < 0.0001
IAI	3 (6.3%)	17 (11.5%)	75 (35.0%)	
IA2	12 (25.0%)	61 (41.2%)	95 (44.4%)	
IA3	8 (16.7%)	21 (14.2%)	19 (8.9%)	
IB	15 (31.2%)	23 (15.5%)	10 (4.7%)	
II	5 (10.4%)	11 (7.4%)	3 (1.4%)	
III	5 (10.4%)	15 (10.2%)	1 (0.5%)	
Histological subtype				
AIS/MIA	0 (0.0%)	2 (1.4%)	29 (13.5%)	*p* < 0.0001
Other	48 (100.0%)	146 (98.6%)	185 (86.5%)	
Operation method				
Lobectomy	36 (75.0%)	119 (80.4%)	131 (61.2%)	*p* = 0.0003
Sublobar resection	12 (25.0%)	29 (19.6%)	83 (38.8%)	

Abbreviations: AIS, adenocarcinoma in situ; CEA, carcinoembryonic antigen; CT, computed tomography; MIA, minimally invasive adenocarcinoma; PYI, pack‐year index; SUVmax, maximum standardized uptake value.

### Relationship between OS and RFS with the combination of SUVmax and CEA

**FIGURE 2:**
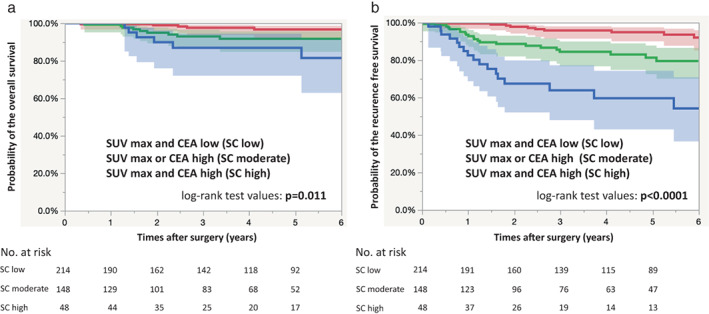
Kaplan–Meier curves showing overall survival (a) and recurrence‐free survival (b) of patients with preoperative clinical stage IA lung adenocarcinoma according to the combination of maximum standardized uptake value (SUVmax) and carcinoembryonic antigen (CEA)

Figure 2 shows the OS and RFS curves for patients in the high SC group, moderate SC group and low SC group. Log‐rank test showed that patients in high SC group had a significantly shorter OS than patients in the moderate and low SC groups (*p* = 0.011; Figure 2a) and patients in the high SC group had a significantly shorter RFS than patients in the moderate and low groups (*p* < 0.0001; Figure 2b). The 5‐year OS rate was 96.7, 91.7 and 86.9% in the low, moderate and high SC groups, respectively. The 5‐year RFS rate was 94.9, 80.2 and 57.2% in the low, moderate and high SC groups, respectively.

Multivariate analysis showed that high value of SUVmax and CEA and age (≥ 65 years) were independent prognostic factors for OS (*p* = 0.020, *p* = 0.0303, respectively) (Table 3), and that high values of SUVmax and CEA were also independent prognostic factors for poor RFS (*p* < 0.0001) (Table 4).

**TABLE 3:** Univariate and multivariate analyses of the overall survival in patients with clinical stage IA lung adenocarcinoma

Factors	Univariate analysis		Multivariate analysis	
	HR (95% CI)	*p*‐value	HR (95% CI)	*p*‐value
Age				
≥65 years	5.232 (1.219–22.461)	0.026	5.033 (1.166–21.722)	0.030
<65 years	1		1	
Sex				
Male	1.243 (0.537–2.880)	0.611		
Female	1			
Smoking status				
<20 PYI	1.961 (0.847–4.541)	0.116		
≥20 PYI	1			
C/T ratio				
≥0.5	1.128 (0.416–3.508)	0.813		
<0.5	1			
Operation method				
Sublobar resection	1.682 (0.717–3.945)	0.232		
Lobectomy	1			
Clinical stage				
IA3	1.594 (0.486–5.229)	0.584		
IA2	1.663 (0.615–4.500)			
IA1	1			
SUVmax and CEA				
High	4.924 (1.587–15.282)	0.020	4.400 (1.415–13.679)	0.029
Moderate	2.708 (0.983–7.456)		2.844 (1.032–7.838)	
Low	1		1	

Abbreviations: CEA, carcinoembryonic antigen; CI, confidence interval; C/T, consolidation/tumor; HR: hazard ratio; PYI, pack‐year index; SUVmax, maximum standardized uptake value.

**TABLE 4:** Univariate and multivariate analyses of the recurrence‐free survival in patients with clinical stage IA lung adenocarcinoma

Factors	Univariate analysis		Multivariate analysis	
	HR (95% CI)	*p*‐value	HR (95% CI)	*p*‐value
Age				
≥65 years	1.791 (0.915–3.506)	0.089		
<65 years	1			
Sex				
Male	1.468 (0.837–2.574)	0.181		
Female	1			
Smoking status				
<20 PYI	1.278 (0.731–2.234)	0.390		
≥20 PYI	1			
C/T ratio				
≥0.5	3.096 (1.229–7.802)	0.005		
<0.5	1			
Operation method				
Sublobar resection	1.044 (0.570–1.912)	0.890		
Lobectomy	1			
Clinical stage				
IA3	5.879 (2.483–13.920)	0.0004		
IA2	2.985 (1.280–6.958)			
IA1	1			
SUVmax and CEA				
High	9.891 (4.521–21.640)	<0.0001	9.891 (4.521–21.640)	<0.0001
Moderate	3.754 (1.786–7.890)		3.754 (1.786–7.890)	
Low	1		1	

Abbreviations: CEA, carcinoembryonic antigen; CI, confidence interval; C/T, consolidation/tumor; HR: hazard ratio; PYI, pack‐year index; SUVmax, maximum standardized uptake value.

### Subset analyses of lobectomy and segmentectomy groups

In the analysis of the lobectomy group, there was no significant difference in OS between the SC high, moderate and low groups (*p* = 0.3177; Figure S1a); patients in the high SC group had a significantly shorter RFS than patients in the moderate and low SC groups (*p* = 0.0003; Figure S1b). In the segmentectomy group, patients in the high SC group had a significantly shorter OS and RFS than patients in the moderate and low groups (*p* = 0.0027; Figure 2a, *p* < 0.0001; Figure 2b, respectively).

### Subset analyses of the relationship between operation method and recurrence rate

We investigated the relationship between operation method and recurrence rate. The recurrence rate tended to be higher in patients in the high SC group (*p* = 0.0553) (Table S1a**)** than in those in the moderate and low groups (*p* = 0.4366) (Table S1b), especially in the sublobar resection group. These findings suggest that the operative method tended to have a relationship with the recurrence rate in the high SC group patients.

## DISCUSSION

In this study, we examined the prognostic significance of the combination of SUVmax and CEA in stage IA lung adenocarcinoma. The Kaplan–Meier curve analysis for OS and RFS showed that high SC was associated with poor prognosis in patients with stage IA lung adenocarcinoma. Moreover, multivariate analysis showed that high SC was an independent predictive factor of a poor OS and RFS. Clinical stage was not an independent prognostic factor; therefore, the combination of SUVmax and CEA may be a more useful prognostic factor than clinical stage.

In the high SC group, more patients who underwent a reduction surgery had recurrences than those who underwent lobectomy (Table S1). In addition, in the analysis of the lobectomy group, there was no significant difference in OS between the SC groups, although patients in high SC group had a significantly shorter OS than patients in the moderate and low SC groups in the sublobar resection group. Together, these findings suggest that lobectomy may be the optimal operation method for patients with clinical stage IA lung adenocarcinoma in the high SC group. Therefore, assessment of preoperative SUVmax and CEA might be important in patients with small sized lung adenocarcinoma for determining the optimal treatment strategy, such as operation method.

CEA measurement is a noninvasive diagnostic tool that is commonly used for cancer screening. Increased levels of CEA in serum occurs is approximately 35%–60% of NSCLC patients.^19^ CEA enhances cancer metastasis through its function as a chemoattractant and an adhesion molecule,^20^ which is associated with poor prognosis in many carcinomas. A preoperative high CEA level has been reported to be a significantly poor prognostic factor in clinical stage IA lung adenocarcinoma.^13^, ^14^, ^21^ CEA has previously been reported to be a clinical predictor of tumor invasiveness and lymph node metastases.^22^ In addition, a systematic review showed that high CEA level is associated with high rates of lymph node involvement and high mortality.^16^

A recent meta‐analysis showed that high SUVmax measured by PET‐CT predicted a higher risk of recurrence or death in patients with NSCLC. In patients with clinical stage 0–IA lung adenocarcinoma, a high SUVmax correlates with high‐risk pathological features, including visceral pleural involvement, pulmonary metastasis, lymph node involvement, lymphatic permeation and vascular involvement.^5^ In addition, high SUVmax is associated with high programmed cell death‐ligand 1 (PD‐L1) protein expression.^23^ PD‐L1 helps cancer cells evade attack by immune cells^24^ and PD‐L1 expression is a significant poor prognostic factor in patients with lung adenocarcinoma.^25^ Taken together, these results indicate that the high value of SUVmax is a risk factor of poor prognosis of lung adenocarcinoma and an important factor for deciding treatment strategy.

As described above, several studies have shown that high CEA and high SUVmax are each associated with the poor prognosis of NSCLC. However, in clinical practice, these individual factors cannot serve as prognostic markers to determine treatment strategy such as surgical procedures or adjuvant chemotherapy. In the current study, we investigated the relationship between the combined SUVmax plus CEA and prognosis in patients with clinical stage IA lung adenocarcinoma. Our results indicate that compared with high value of SUVmax or high level of CEA alone, high values of both SUVmax and CEA are associated with poor prognosis. Multivariate analysis showed that high value of both SUVmax and CEA was an independent prognostic factor for RFS (HR: 0.274; 95% CI: 0.150–0.502; *p* < 0.0001). Further prospective studies are warranted to evaluate preoperative grading with the combination of SUVmax and CEA for the optimal treatment strategy of clinical stage IA lung adenocarcinoma.

This study had several limitations. First, this was a single‐institution retrospective study. Second, serum CEA levels are often influenced by various factors, such as smoking and aging. Third, SUVmax may be influenced by various factors, such as body composition, habitus, length of uptake period, plasma glucose and recovery coefficient and partial volume effects.^26^

In conclusion, our findings showed that high value of both SUVmax and CEA was associated with poor prognosis in patients with stage IA lung adenocarcinoma and was an independent factor of short RFS.

## CONFLICT OF INTEREST

All authors declare no conflicts of interest associated with this research.

## Supporting information


**Table S1** Association of the presence or absence of recurrence with operation method in patients with high SUVmax and CEA group (a) and moderate and low SUVmax and CEA group (b)
**Figure S1**. Kaplan–Meier curves showing overall survival (a) and recurrence‐free survival (b) of patients with preoperative clinical stage IA lung adenocarcinoma who underwent lobectomy according to the combination of SUVmax and CEA.
**Figure S2**. Kaplan–Meier curves showing overall survival (a) and recurrence‐free survival (b) of patients who underwent sublobar resection.
