# Supplementary material for: Clinical significance of the combination of preoperative SUVmax and CEA in patients with clinical stage IA lung adenocarcinoma
Source: Thorac Cancer. 2022 Aug 12;13(18):2624–32. doi: 10.1111/1759-7714.14599 (PMC9475234; doi:10.1111/1759-7714.14599)
Supplement: Supplementary file 1 — Table S1 Association of the presence or absence of recurrence with operation method in patients with high SUVmax and CEA group (a) and moderate and low SUVmax and CEA group (b) Figure S1. Kaplan–Meier curves showing overall survival (a) and recurrence‐free survival (b) of patients with preoperative clinical stage IA lung adenocarcinoma who underwent lobectomy according to the combination of SUVmax and CEA. Figure S2. Kaplan–Meier curves showing overall survival (a) and recurrence‐free survival (b) of patients who underwent sublobar resection. [file TCA-13-2624-s001.docx]

**Supplemental table 1**. Association of the presence or absence of recurrence with operation method in patients with high SUV max and CEA group (A) and moderate and low SUV max and CEA group (B)

(A)

| **Factors** |  | **Recurrence (+) n=17** | **Recurrence (-) n=31** | **p value** |
| --- | --- | --- | --- | --- |
| Operation  method | Lobectomy | 10 (27.8%) | 26 (72.2%) | p=0.0553 |
|  | Sublobar resection | 7 (58.3%) | 5 (41.7%) |  |

(B)

| **Factors** |  | **Recurrence (+) n=25** | **Recurrence (-) n=337** | **p value** |
| --- | --- | --- | --- | --- |
| Operation  method | Lobectomy | 19 (7.6%) | 231 (92.4%) | p=0.4366 |
|  | Sublobar resection | 6 (5.4%) | 106 (94.6%) |  |

Abbreviations: CEA, carcinoembryonic antigen; SUV, standardized uptake value

**Supplemental figure 1**. Kaplan-Meier curves showing overall survival (A) and recurrence-free survival (B) of patients with preoperative clinical stage IA lung adenocarcinoma who underwent lobectomy according to the combination of SUV max and CEA.

**Supplemental figure 2**. Kaplan-Meier curves showing overall survival (A) and recurrence-free survival (B) of patients who underwent sublobar resection.
